# *In Vitro* Lethal Activity of the Nematophagous Fungus* Clonostachys rosea* (Ascomycota: Hypocreales) against Nematodes of Five Different Taxa

**DOI:** 10.1155/2018/3501827

**Published:** 2018-03-20

**Authors:** Rosalia Rodríguez-Martínez, Pedro Mendoza-de-Gives, Liliana Aguilar-Marcelino, María Eugenia López-Arellano, Marcela Gamboa-Angulo, Greta Hanako Rosas-Saito, Manuela Reyes-Estébanez, Virginia Guadalupe García-Rubio

**Affiliations:** ^1^Área de Helmintología, CENID-Parasitología Veterinaria, INIFAP, Carretera Federal Cuernavaca-Cuautla No. 8534, Col. Progreso, 62500 Jiutepec, MOR, Mexico; ^2^Facultad de Medicina Veterinaria y Zootecnia, Universidad Autónoma del Estado de México, Carretera Amecameca-Ayapango, Km 2.5, 56700 Toluca de Lerdo, MEX, Mexico; ^3^Unidad de Biotecnología, Centro de Investigación Científica de Yucatán (CICY-CONACYT), Chuburná de Hidalgo, No. 130, 972005 Mérida, YUC, Mexico; ^4^INECOL, A.C. Red de Estudios Moleculares Avanzados, Unidad de Microscopía Avanzada del Clústec Científico y Tecnológico Biomimic, Carretera Antigua a Coatepec No. 351, Col. El Haya, 91070 Xalapa, VER, Mexico; ^5^Universidad Autónoma de Campeche, Calle Avenida Agustín Melgar s/n, Buenavista, 24039 Campeche, CAM, Mexico

## Abstract

This study was aimed to evaluate the* in vitro* lethal activity of the nematophagous fungi* Clonostachys rosea* against 5 nematodes species belonging to different taxa. Two groups of 35 Petri dishes (PD) each were divided into 5 series of 7 (PD). Group 1 (series 1, 2, 3, 4, and 5) contained only water agar; meanwhile group 2 plates (series 6, 7, 8, 9, and 10) contained* C. rosea* cultures growth on water agar. Every plate from the two groups was added with 500 nematodes corresponding to the following genera/specie:* Haemonchus contortus*,* Caenorhabditis elegans, Rhabditis* sp.,* Panagrellus redivivus*, and* Butlerius* sp. After 5-day incubation at room temperature, free (nontrapped) larvae were recovered from plates using the Baermann funnel technique. Recovered nematodes were counted and compared with their proper controls. Results shown an important reduction percentage of the nematode population attributed to the fungal lethal activity as follows:* H. contortus *(L_3_) 87.7%; C.* elegans *94.7%;* Rhabditis *sp. 71.9%;* P. redivivus *92.7%; and* Butlerius *sp. 100% (*p* ≤ 0.05). The activity showed by* C. rosea* against the* H. contortus* can be crucial for further studies focused to the biological control of sheep haemonchosis, although the environmental impact against beneficial nematodes should be evaluated.

## 1. Introduction

**Figure 1:**
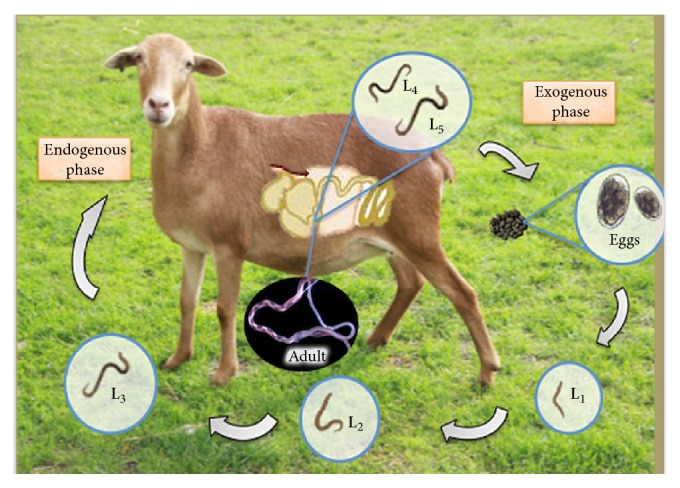
Life cycle of* Haemonchus contortus*.

Gastrointestinal parasitic nematodiases (GPN) are a group of parasitosis affecting the livestock industry and their economic consequences are considered one of the major concerns for producers all over the world [1]. Yield losses caused by GPN in Mexico have been recently estimated to be US$ millions 445.10 per year [2]. The species* Haemonchus contortus* is considered the most pathogenic parasite causing the lack of growth, malnutrition, low feeding conversion, appetite loss, and even the death of young animals [3, 4]. A survey carried out in Australia revealed economic losses caused by* H. contortus* in sheep by AUD$ 436 million dollars a year [5]. Farmers have to resort to the use of chemical anthelmintic drugs (AD) to diminish the health havocs in their flocks and their economic consequences [4, 6]. The use of AD is the method most commonly used to control sheep parasitosis; however, the imminent presence of anthelmintic resistance in the parasites provokes that most of AD are dramatically losing their deworming efficacy [7]. Different alternatives to control GPN other than the use of AD have been searched during the last decades [8]. The potential use of natural-nematode antagonists against GPN of cattle and small ruminants as a biotechnological tool is being developed in some countries around the world [9–13]. Natural-nematode enemies act as bioregulator agents of nematode populations in soil. The life cycle of* H. contortus* includes two stages: one occurs in the gastrointestinal tract of animals; the other stage takes place outside their host on the feces and pasture as eggs and larvae, where they are incorporated into the environment as free-living nematodes [14] (Figure 1).

In this stage, a close relationship is established with a wide variety of other soil nematodes, belonging to different taxa such as* Caenorhabditis elegans, Rhabditis* sp.,* Panagrellus redivivus*, and* Butlerius* sp. These free-living nematodes and a wide variety of soil nematodes play an important role in different biological and ecological processes. These nematodes as a whole miscellaneous population share the same habitat soil with a number of different microorganisms; some of which act as natural predators of nematodes, that is, nematophagous fungi such as* Duddingtonia flagrans* [9] and* Clonostachys rosea* [15, 16]. Nematophagous fungi are a group of microfungi living in soil and they have the capacity to differentiate their mycelia in trapping devices to capture, destroy, and feed on soil nematodes acting as the main bioregulators of the nematode population in soil [9]. The use of these fungi has been considered as a sustainable control strategy of nematodes of cattle [12] and sheep [17]. The hypocreal fungus* C. rosea *inhabits soil as part of the natural mycobiota. This fungus has been identified as an endoparasite nematode, producing resistance spores that attach to the nematode cuticle, or they can be ingested by nematodes [15]. On the other hand,* Duddingtonia flagrans* produces three-dimensional adhesive rings where nematodes are captured and destroyed following the same process of* C. rosea *[16]. Both fungi are promising biotechnological tools of control of ruminant parasitic nematodes, although it is crucial to get more information about their activity against soil nematodes belonging to different taxa. The present research was aimed at evaluating the* in vitro* lethal activity of* C. rosea* against five different genera/specie nematodes including* H. contortus *(L_3_) and different development stages of* C. elegans, Rhabditis* sp.,* P. redivivus*, and* Butlerius *sp.

## 2. Materials and Methods

This study was carried out at the National Centre for Research in Veterinary Parasitology (CENID-PAVET), from the National Institute for Forestry, Agriculture and Livestock Research (INIFAP-MEXICO). This center is situated at Jiutepec Municipality, State of Morelos, Mexico. This subtropical location is at 1,350 meters over sea level and has a subhumid climate where precipitation ranges within 800–1400 mm with a yearly average temperature of 20–24°C. Maximum precipitation occurs from June to September, the minimum from December to May [18].

### 2.1. Production of* Clonostachys rosea*

A* C. rosea* strain (TH27) belonging to the Centro de Investigación Científica de Yucatán (CICY-CONACYT) was used. This strain was originally isolated from submerged leaves from “cenote Temozon,” an aquatic ecosystem, at the Ecological Reserve called “Parque Eco-Arqueológico Dzibilchaltún” at Mérida, Yucatán, Mexico. Leaves were incubated at room temperature in damp chambers to induce sporulation which were isolated and purified in potato-dextrose agar. For the massive spore production,* C. rosea* was cultured in PDA and maintained at 25°C, 12/12 h light/darkness for seven days [19]. Spores of this strain were preserved by lyophilization [20, 21].

### 2.2. Nematode Production

#### 2.2.1. *Haemonchus contortus*

A Mexican strain of* H. contortus* originally obtained from a clinical case of sheep haemonchosis from a farm in Hueytamalco, State of Puebla, Mexico, was used. Female nematodes were collected from abomasum at the necropsy of the animal and they were dissected to remove their gravid uterus to eventually obtain their eggs. Eggs were processed following the technique described by [22] to promote the embryo development into the eggs and to get a quite large amount of active larvae. Infective larvae of this parasite were used to artificially infect a young sheep with 350 L3 of the parasite per Kg of body weight [23]. The artificially infected sheep was used as an egg donor and once sheep was diagnosed as positive to the presence of nematode eggs, feces were collected and fecal cultures in plastic bowls were performed by grinding and polystyrene particles and top water were added and a mixture was achieved to provide a suitable humid and oxygen atmosphere to assure the most possible larvae recovering. After 5- to 10-day incubation, most of larvae developed until reaching the infective stage and they were recovered through the Baermann technique for 12 h [23].

#### 2.2.2. *Panagrellus redivivus*

A* P. redivivus* strain belonging to the National Metropolitan Autonomous University (Campus Xochimilco, Mexico) was donated for our study. Free-living nematodes were cultured using commercial oat flakes and purified water mixed into plastic bowls. Ingredients were sterilized into a microwave oven for five minutes. Bowls were covered with foil with a small window protected with gauze to allow the gas interchange (Oxygenation) and avoid the insect entrance and contamination. Fecal cultures remained at room temperature for 25 days [24].

#### 2.2.3. *Rhabditis* sp.

A* Rhabditis* sp. strain was isolated from a soil sample from Jiutepec Municipality, Morelos State, Mexico. Soil samples were deposited on water agar plates and incubated at room temperature (18–25°C). The presence of free-living nematodes sliding on the agar surface was observed after 7-day incubation. Nematodes were transferred to 2% sterile water agar plates and one g of peanut butter was added on the center of the plates as a source of energy to promote a large nematode population growing. Nematodes were transferred every 20 days to fresh culture media to maintain a continuous nematode production [25].

#### 2.2.4. *Caenorhabditis elegans*

A* C. elegans* strain was kindly supplied by the Institute of Biotechnology, National Autonomous University of Mexico (UNAM), maintained by passes to NGM (Nematode Growing Medium) with an* Escherichia coli* wild-type strain, and incubated at 37°C for 24 h [26].

#### 2.2.5. *Butlerius* sp.

An isolate of the predatory nematode classified into the genus* Butlerius *sp. was obtained from a garden soil sample amended with compost material from the Huitzilac Municipality, Morelos State, Mexico. The isolation of soil predatory nematodes was performed following the Whitehead and Hemming tray technique [27]. Recovered nematodes were maintained in water agar plates added with free-living nematodes of the genus* Rhabditis *sp. at room temperature (25–29°C). Nematodes were observed under a microscope to identify the ones feeding on the free-living nematodes. The taxonomic identification of free-living and predatory nematodes was performed based on the descriptions published by [28–32]. Predatory nematodes were maintained on water agar plates amended with an unknown number of specimens of the free-living nematodes and 1 g of peanut butter was deposited in the center of the agar plates as an additional source of energy to promote the development of bacteria in benefit of both kinds of nematodes.

Fecal cultures were elaborated using grinded sterile sheep feces mixed with small particles of polyurethane and tap water into a plastic bowl. An undetermined number of* Butlerius* sp. specimens were added and also an unknown amount of* P. redivivus* was added as a source of food for nematodes. Finally a piece of foil was used to cover the fecal cultures and a 10 cm^2^ window with a gauze was adapted to the foil allowing oxygen interchange and to promote a suitable development of eggs/larvae. Fecal cultures were maintained at 18–25°C temperature for 25 days [30, 33, 34].

### 2.3. Experimental Design

#### 2.3.1. Predatory/Prey Nematode Confrontation Conditions

Two series of 35 plastic Petri dishes (60 × 15 cm) containing 2% sterile bacteriological water agar were established. Series 1 was considered as the control series and included five subseries of seven plates each. Subseries (series 1) were designed as 1, 2, 3, 4, and 5 subseries and 500 nematodes of the following specimens* H. contortus* L_3_,* Rhabditis* sp.,* C. elegans*,* P. redivivus*, and* Butlerius* sp. were added to every plate; respectively. Subseries in Series 2 were designed as 6, 7, 8, 9, and 10 subseries. This series contained the same number of plates, same medium, and same nematode species and was maintained exactly under the same conditions as in series 1, with the only difference being that plates in this series contained a seven-day-old* C. rosea *culture, and it was considered as the treated series. The confrontation time was five days at room temperature (25–30°C).

#### 2.3.2. Nematode Recovering

After confrontation period the whole content of every plate from every series was transferred to an individual Baermann funnel and were remained for 24 h allowing the nematode migration to the base of assay tubes. Nematodes were recovered from sediment and quantified. Ten 5 *μ*L aliquot drop were taken and deposited on slides and larvae were observed and counted using a light microscope (×10). Nematode reduction percentage attributable to the fungal action was estimated using the following formula:

(1) $$\begin{array}{c} A=\frac{X\;\;control-X\;\;treated}{X\;\;control}*100, \end{array}$$

where *X* control is mean of recovered nematodes from control series; *X* treated is mean of recovered nematodes from treated series, [25].

### 2.4. Statistical Analysis

Data were analysed using a completely random design, considering the means of recovered nematodes as the dependent variable. An ANOVA analysis was used followed by Tukey's test as a complementary analysis. Data were analysed using the Statistical Analysis System (SAS) software [35].

## 3. Results and Discussion

**Figure 2:**
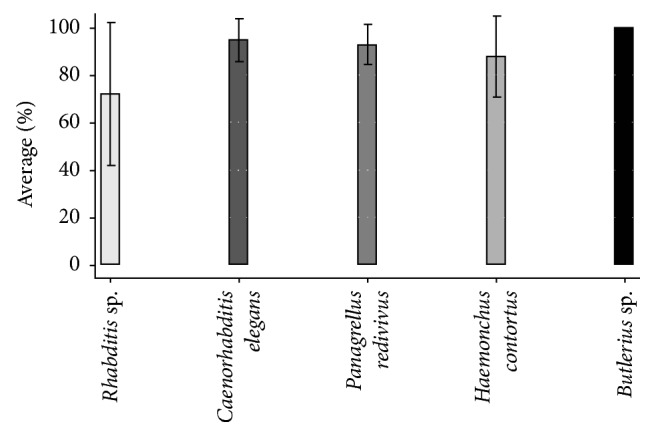
Means of recovered nematodes after five days interaction with the nematophagous fungus* Clonostachys rosea* (*p* ≤ 0.05).

Means and standard deviation of nematode recovery by genus/species from replicates in both series (control and treated) are shown in Table 1. Reduction percentages of the different nematodes confronted with the fungi are shown in Figure 2. Reduction percentages recorded in the different nematodes were as follows: 71.9% for* Rhabditis* sp.; 94.7% for* C. elegans; *92.7% for* P. redivivus*; 87.7% for* H. contortus*; and 100% for* Butlerius* sp. The comparison of means showed that nematode reduction values attributable to the fungal action were significant (*p* ≤ 0.05). Nematode reduction means for* C. elegans*,* P. redivivus*, and* H. contortus* did not show differences among subseries. However, differences between subseries with* Rhabditis* sp. and* Butlerius *sp. were observed (*p* ≤ 0.05).

**Figure 3:**
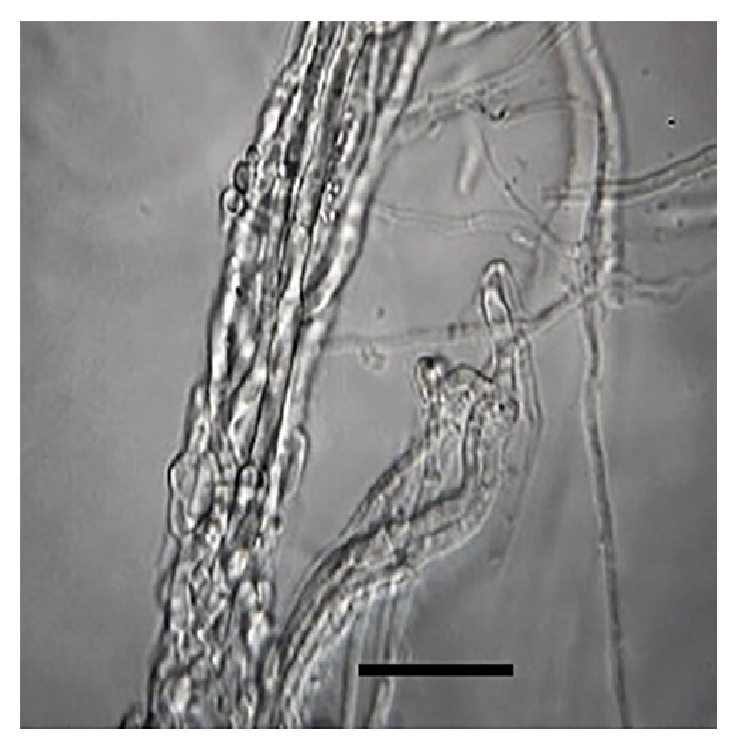
*Haemonchus contortus* infective larvae emptied carcass filled with* Clonostachys rose *mycelia after five days in vitro interaction (100x magnification) bar = 20 *μ*m.

The present research showed evidence of a high* in vitro* predatory activity of* C. rosea* against five different nematode genera/species. The lethal fungal activity of* C. rosea* against the different prey nematodes after five-day interaction ranged between 71.9 and 100%,* Butlerius* sp. being the most susceptible prey compared to the other assessed nematodes. On the other hand, free-living nematodes* C. elegans* and* P. redivivus *and the sheep parasitic nematode* H. contortus* (L_3_) showed a little lower susceptibility to be preyed than* Butlerius* sp. by* C. rosea *(Figure 3). The lowest fungal predatory activity (72%) was observed against the free-living nematode* Rhabditis* sp., although the statistical analysis revealed that there was no difference in the fungal activity against the different assessed nematodes.

The results of the present study showed that* C. rosea* acted as a voracious nematode predatory fungus of different taxonomic groups, despite the kind of nematode prey. The fact that* C. rosea* showed an important* in vitro* predatory activity against* H. contortus* is a desirable feature, since this strain could be a good candidate for future studies focused to explore its antagonistic potential as a biological control agent of sheep haemonchosis. On the other hand, the fact that* C. rosea* showed a strong predatory activity against the three assessed free-living nematodes seems to be undesirable, since free-living nematodes are beneficial microorganisms playing an important role in soil as nitrogen cycling microorganisms [36]. Despite this, free-living nematode populations in soil possess an enormous capability to reproduce themselves being one of the most prolific organisms living in soil. The numbers of nematodes in microbiologically active soil are estimated to range from one to twenty million individuals per square meter [37]. So, no important decreasing in the free-living nematode population would be expected under field conditions, although this is only a speculation that should be proved. In contrast, due to its different life cycle,* H. contortus* has a totally different growing population pattern where infective larvae need their ruminant hosts to complete their life cycle. So, there is no chance to increase the population of* H. contortus* in soil without the presence of their ruminant hosts. On this way, the propose of reducing the* H. contortus* infective larvae population in soil/grass as an indirect method of control by the* C. rosea *effect could be expected by using* C. rosea* as a biological control agent.

In addition to the predatory mechanical activity of* C. rosea* hyphae against nematodes a chemical activity of this and other nematophagous fungi has been found as a complementary strategy to destroy and to feed on nematodes in nature. In this context, an intense fungal chemical activity through enzyme and secondary metabolite production are being identified and they possess a nematicidal effect against several kind of nematode species. The fungus* C. rosea* has been evaluated to identify the nematicidal effect of a number of metabolites obtained through chromatographic processes and they are being identified as follows: Gliocladine A, B, C, D, and E. These metabolites showed 50% lethal activity after 24 h against three different nematode genera/species:* C. elegans, P. redivivus*, and* Bursaphelenchus xylophilus *[38]. More recently, a study about a high nematostatic activity (>90%) of a group of* C. rosea* biocompound obtained from chromatographic processes against* H. contortus* was published [39]. On the other hand, an antibacterial, antifungal, and insecticide bioactive compound was obtained from* Chaetosphaeria cylindrospora*. Such compound was called “Clonostachydiol” and it was classified into the macrodiolids group. In addition, such biocompound possesses an important nematicidal activity against* H. contortus* when is subcutaneously administered at 2.5 mg/kg BW producing 80 to 90% parasitic burden reduction [40]. In the present research, no chemical study was performed; however, the* C. rosea *strain could be a good candidate to search for metabolites with nematicidal activity against* H. contortus* and other ruminant parasitic nematodes of importance for livestock industry.

## 4. Conclusions

The results of the present study show a high* in vitro* predatory activity of* C. rosea* against* H. contortus* infective larvae and it will be considered in future research works focused to find a practical use of this fungus in the control of sheep haemonchosis and other nematodiasis affecting sheep industry.

## Figures and Tables

**Table 1:** Individual reduction percentage of five different nematode genera/species by the predatory activity of the nematophagous fungi *Clonostachys rosea* after five-day *in vitro* confrontation.

Nematodes					
Replicate	*Rhabditis* sp.^*∗*^	*Caenorhabditis elegans* ^*∗*^	*Panagrellus redivivus* ^*∗*^	*Haemonchus contortus* (L_3_)	*Butlerius* sp.^*∗*^
(1)	40	100	95.8	85.7	100
(2)	100	100	90	100	100
(3)	33.3	80	100	61.5	100
(4)	50	100	100	66.6	100
(5)	100	100	82.3	100	100
(6)	100	100	100	100	100
(7)	80	83,3	80.9	100	100

Σ =	503.3	663.3	649.1	613.9	700
Mean	**71.9**	**94.7**	**92.7**	**87.7**	**100**
SD =	30.0	9.0	8.3	17.0	0

*p* ≤ 0.05; ^*∗*^a mixture of developing stages.
